# Fractal correlation properties of HRV as a noninvasive biomarker to assess the physiological status of triathletes during simulated warm-up sessions at low exercise intensity: a pilot study

**DOI:** 10.1186/s13102-022-00596-x

**Published:** 2022-12-01

**Authors:** Marcelle Schaffarczyk, Bruce Rogers, Rüdiger Reer, Thomas Gronwald

**Affiliations:** 1 Institute of Interdisciplinary Exercise Science and Sports Medicine, MSH Medical School Hamburg, University of Applied Sciences and Medical University, Am Kaiserkai 1, 20457 Hamburg, Germany; 2 Department of Internal Medicine, College of Medicine, University of Central Florida, Orlando, USA; 3 Department Sports and Exercise Medicine, Institute of Human Movement Science, University of Hamburg, Hamburg, Germany

**Keywords:** DFA a1, Endurance exercise, Fatigue, Monitoring

## Abstract

**Background:**

The non-linear index alpha 1 of Detrended Fluctuation Analysis (DFA a1) of heart rate variability, has been shown to be a marker of fatigue during endurance exercise. This report aims to explore its ability to assess the physiological status as a surrogate metric for “readiness to train” while performing simulated warm-up sessions the day after two different exercise sessions.

**Methods:**

11 triathletes were recruited to determine the first ventilatory threshold (VT1) during a baseline assessment and to perform 10-min of cycling at 90% of VT1 (simulating a warm-up bout) before (PRE) and within 36 h after (POST) light and heavy running exercise. RR intervals were recorded for DFA a1 analysis along with neuromuscular testing to verify the effects of the performed exercise sessions. In addition to common statistical methods, magnitude-based inferences (MBI) were applied to assess the changes in true score and thus also the practical relevance of the magnitude.

**Results:**

Rating of perceived exertion for the heavy exercise session showed a significant higher rating as opposed to the light exercise session (*p* < 0.001, d = 0.89). In regard of MBIs, PRE versus POST comparisons revealed a significant reduced DFA a1 with large effect size after the heavy exercise session (*p* = 0.001, d = − 1.44) and a 99% chance that this negative change was clinically relevant.

**Conclusions:**

Despite inter-individual differences, DFA a1 offers potential to assess physiological status and guide athletes in their training as an easy-to-apply monitoring procedure during a standardized warm-up. A regular assessment including individual data history and statistical references for identification of response is recommended. Further data are necessary to confirm the results in a larger and more homogeneous population.

## Introduction

Numerous studies have been conducted to investigate what is the optimal upcoming daily training plan in terms of balancing exercise loads (e.g. exercise intensity and volume) and proper recovery [e.g. 1–4]. Some evidence even suggests that daily directed training decisions based on certain physiologic metrics can lead to longer term performance improvements [5–7]. This monitoring-based decision-making requires an individual approach [8].

In this context, the term "readiness to train" is often used to describe this process, but there is still a lack of both a universal understanding of this concept, its measurability, and validity [9]. Descriptions of this concept include, the absence of signs of impaired physical performance, mental fatigue or excessive psychological distress and “the athlete’s capacity to complete training activities and perform during competition” [8]. Systemic readiness is most commonly assessed through whole-body neuromuscular function as a marker of fatigue as well as by longitudinal monitoring of chronic fitness adaptations [10]. It is expected that neuromuscular performance (e.g. Counter Movement Jump (CMJ), repeated rebound jumps, sprint testing) worsens with increases in fatigue which is the response to workload alterations [10]. Since it is claimed that “readiness” has also physical, physiological and psychosocial underpinnings [10], monitoring options may also include measures about metabolic (e.g. blood lactate), psychological (e.g. psychometric scales, rating of perceived exertion, RPE) and autonomic status with heart rate (HR) variability (HRV) being attributed to the last group [4]. There are promising results for HRV indices (e.g. high frequency (HF) power, root mean square of successive differences (RMSSD)) with regard to functional overreaching [11], autonomic recovery status [1, 12] or training status and “readiness to perform” [13] when applied during rest or post exercise conditions [1, 11, 12] allowing one to select an appropriate training effort based on a proxy of the regulation balance associated with the autonomic nervous system (ANS). However, studies recommend the parallel use of specific physiological markers with daily training logs and/or psychometric short scales to take full advantage of such an approach [1, 14, 15]. Although seemingly simple at the surface, studies report large day-to-day variations in isolated resting HRV measures (most studied time-domain metric: RMSSD) due to multiple influencing factors like environmental conditions [16], exercise induced changes in blood plasma volume [17] or the presence of residual (acute) fatigue from previous training sessions [18]. From the logistical standpoint, resting HRV requires a regular day-to-day monitoring routine including standardization (e.g., time of day, body position; [19]). Since an application of conventional time-domain parameters during endurance exercise is even less informative due to a loss of dynamics past the aerobic threshold [20], alternative approaches for HRV analysis are needed.

A recent HRV metric that has been associated with endurance exercise related fatigue [21] is the non-linear index alpha 1 of Detrended Fluctuation Analysis (DFA a1) representing fractal correlation properties of the beat to beat pattern. Its dynamic range varies between ~ 1.5 mirroring a more correlated or periodic behavior and ~ 0.5 indicating a loss of fractal dynamics and complexity toward a more random behavior (disorganized randomness) [22]. Studies have shown its low dependency on HR [23, 24], as well as its suitability to describe the complex cardiac autonomic regulation during various exercise intensities, modalities, and environmental conditions [25]. While higher values were attributed to a reintegration and synchronization of subsystems, lower values were supposed to indicate a disintegrating and centralization process [22, 25]. In addition to its piloting usage for detecting endurance exercise fatigue it has also been explored as a delimiter for physiological thresholds during incremental exercise protocols [26–29]. In one fatigue-related study DFA a1 was analyzed before and after an ultramarathon in an exercise group and respectively pre and post daily activity in a control group [21]. DFA a1 exhibited suppressed behavior only in the ultramarathon group during constant low intensity exercise at around the aerobic threshold, post ultramarathon, associated with reduced CMJ scores indicating neuromuscular fatigue. These results are consistent with the assumption that DFA a1 is associated with total organismic demands including comprehensive organizational approaches such as the Network Physiology of Exercise (NPE) [30]. This programmatic approach aids to understand exercise-related phenomena (e.g. sports performance, fatigue, overtraining) by compiling multiple neuromuscular, biochemical, peripheral and central nervous system inputs to an overall concept of “organismic demand” [30]. Since this analysis was the first to evaluate DFA a1 as an opportunity for potential real-time tracking of physiological status, further investigation is needed to explore its’ applicability to evaluate the athlete's status in everyday training routines. One example would be an assessment during the warm-up phase of dynamic submaximal exercise to help determine whether autonomic balance is disrupted and thereby leading to adjustments in exercise load. Submaximal fitness tests have been shown to provide a feasible approach to evaluate an athlete’s physiological state (e.g. time-efficiency, low physiological impact, feasibility for implementation) [31]. Such submaximal tests provide a pragmatic approach observing internal load responses in relation to a standardized physical stimulus. Shushan et al. [31] define submaximal tests as “[…] short exercise bouts, undertaken at a standardized intensity that is intended to be non-exhausting, and performed with the purpose of inferring an athlete’s physiological state through the monitoring of relevant outcome measures”. With a prior exercise session (or accumulated workload over multiple sessions) potentially causing some level of fatigue, it would be of interest to see if DFA a1 would show inappropriate suppression, while maintained or elevated values would mirror a positively altered physiological status.

The following report is an initial exploration of the ability of DFA a1 to assess this state during 10-min bouts of cycling at 90% power of the first ventilatory threshold (VT1, representing a warm-up bout) before and after (1) a light and (2) a heavy running exercise session separated by one week.

## Materials and methods

### Participants

Sixteen triathletes were recruited from a local triathlon club. Inclusion criteria comprised healthy men and women in an age range of 18 to 60 with active participation in triathlon and the willingness to attend the 5 planned laboratory visits. Participants were excluded in the case of previous medical history, current medications, and recent illness or with an exceeding artifact number in their data sets. After dropout or rejection of five athletes due to time constraints, injuries or data issues (artifact occurrence rate: > 5%), eleven (ten males, one female) could be included for analysis. They aged 37 ± 10 (range: 21.0–55.0) years and comprised a mean body weight of 72.8 ± 10.4 kg and mean height of 182 ± 8 cm. Triathlon related exercise totaled 9.0 ± 4.4 (range: 3.0–18.0) hours per week. The study was performed in accordance with the Declaration of Helsinki and all participants received the detailed description of the experiment before providing their written informed consent. Ethical approval was obtained from the local ethics committee (University of Hamburg, Department of Psychology and Movement Science, Germany, reference no.: 2021_407).

### Baseline assessment

Each participant was invited to an initial appointment where diagnostics and familiarization of testing routine were performed. An incremental exercise test on a mechanically braked cycle (Ergoselect 4 SN, Ergoline GmbH, Germany) was used to determine the exercise intensity (defined as cycling power in watts (W)) for the further tests and for performance level assessment. The ramp protocol comprised a 3 min ride at 50 W with an increase in power by 1 W every 3.6 s (equivalent to 16.7 W per min). RPE and lactate samples from the capillary blood of the earlobe were taken before start, every 3 min and immediately after stopping the exercise. HR, HRV measures and gas exchange kinetics were recorded continuously with a single channel ECG chest belt device (Movesense Medical sensor, firmware version 2.0.99, Movesense, Finland; sampling rate: 512 Hz; software application: Movesense Showcase app version 1.0.9 for iOS; [32]), as well as with a metabolic analyzer (Quark CPET, Omnia software, version 1.6.5, module A-67-100-02, Cosmed, Italy). The protocol was terminated when the participants could not either hold the predetermined cycling cadence (60–80 rpm) or due to voluntary exhaustion, discomfort or pain. Exhaustion was assumed when the following criteria were fulfilled: (A) heart rate > 90% of the maximum predicted heart rate (prediction model according to Tanaka et al. [33]: 208 − (0.7 × age) and (B) respiratory quotient > 1.15 [34]. Maximum oxygen uptake (VO_2max_) and maximum HR (HR_max_) were defined as the average VO_2_ and HR over the last 30 s of the test. For maximum power (P_max_) the highest observed value was considered.

Determination of the VT1 was made based on the approach of [26]. Thus, oxygen uptake (VO_2_), carbon dioxide (VCO_2_), end-tidal oxygen concentration (PetO_2_), end-tidal carbon dioxide concentration (PetCO_2_) and minute ventilation (VE) were plotted to apply modified V-slope method, ventilatory equivalencies, excess CO_2_ production and PetO_2_ nadir. The first three procedures were based on the recommendations of Gaskill et al. [35] and the last one was suggested by Binder et al. [36]. Power at VT1 was defined as the instantaneous (non-averaged) cycling power reached at that time with 90% thereof prescribing the intensity for follow up DFA a1 testing during the 10-min cycling protocols intended to represent a traditional warm-up. Data resulting from these baseline assessments can be found in Table 1. Familiarization was performed for the upcoming follow-up sessions including the explanation of the further course and the applied methods. The athletes had the opportunity to ask questions and practice on the ground reaction force platform.

**Table 1:** Demographic data and data from the baseline assessment of all included participants (n = 11)

	Sex	Age (years)	Body weight (kg)	Height (cm)	Training volume (h week^−1^)	VO_2max_ (ml kg^−1^ min^−1^)	P_max_ (W)	HR_max_ (bpm)	VO_2_ VT1 (ml kg^−1 ^min^−1^)	VT1 (W)	90% VT1 (W)
1	M	31	57.6	165	8.0	51.2	252	197.0	35.4	142	128
2	M	48	81.0	186	12.0	41.7	362	176.0	32.3	220	198
3	M	43	75.0	180	6.0	54.7	346	177.0	35.8	202	182
4	M	45	65.5	172	10.0	55.4	338	171.0	39.5	210	189
5	M	21	61.0	173	18.0	62.0	342	195.0	41.5	202	182
6	M	39	67.3	185	13.0	50.9	288	160.0	35.3	194	175
7	M	31	87.1	183	12.0	48.5	354	172.0	32.0	220	198
8	M	55	87.5	191	5.0	46.5	390	166.0	33.1	246	221
9	M	36	64.3	185	6.0	51.2	316	177.0	29.5	154	139
10	F	24	73.4	188	3.0	40.7	266	186.0	31.8	186	167
11	M	34	80.6	189	6.0	55.2	400	179.0	33.2	210	189
Mean ± SD	–	37 ± 10	72.8 ± 10.4	182 ± 8	9.0 ± 4.4	50.8 ± 6.2	332 ± 48	177.8 ± 11.3	34.5 ± 3.5	199 ± 30	179 ± 27

VO_2max_, maximum oxygen uptake; P_max_, maximum power; HR_max_, maximum heart rate; VO_2_ VT1, oxygen uptake at first ventilatory threshold; VT1, first ventilatory threshold

### Follow-up study design

**Fig. 1:**
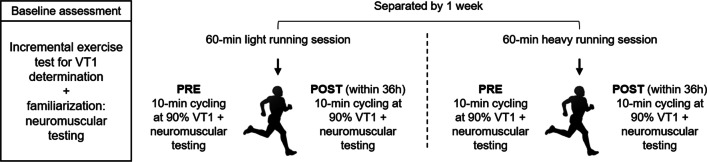
An overview of the study design consisting of the baseline assessment and the follow-up testing

Triathletes were tested on four other occasions: immediately before (PRE) and within 36 h after (POST) (1) a light running session and, (2) a heavy running session separated by one week. The laboratory visits included a designed monitoring test battery of approximately 30 min’ duration consisting of psychometrics, DFA a1 recording during a 10-min low intensity warm-up session at 90% power of VT1 and neuromuscular testing. The training content was designed by the triathletes’ coach in mutual agreement with our research group (Table 2). While the heavy exercise session contained 24 min in a nearly maximal intensity range (95% HR_max_), there were just two intensity peaks of 80% HR_max_ in the light exercise session. Internal workload of these sessions was also assessed by means of a category-ratio “rating of perceived exertion” (RPE) scale from 0 to 10 [37] queried after completed training, so that it could be assessed whether the exercise sessions had fulfilled their purpose of light or heavy impact. Since both sessions comprised a duration of 60 min, multiplication with session duration was neglected. An overview of the study design is shown in Fig. 1.

**Table 2:** Training schedule of the performed sessions controlled by percent of maximum heart rate (%HR_max_)

	Warm-up	Basic endurance	Peak	Rest	Peak	Cool-down
*Light running exercise session*						
Time [min]	10	20	2	2	2	24
Intensity [%HR_max_]	65%	70%	80%	65%	80%	70%

	Warm-up	Basic endurance	Interval (6x)	Rest (6x)	Cool-down	–
*Heavy running exercise session*						
Time [min]	10	5	4	2	9	–
Intensity [%HR_max_]	65%	70%	95%	Walk/trot	65%	–

Upon arrival, the participant was asked to complete a psychometric short questionnaire named Short Recovery and Stress Scale (SRSS) endorsed by studies due to its economic, multidimensional and sensitive properties [38–40]. Eight subscales should be rated with a seven-point Likert-type scale ranging from 0 (“does not apply at all”) to 6 (“fully applies”). These include: *physical performance capability* (PPC), *mental performance capability* (MPC), *emotional balance* (EB), *general overall recovery* (OR), *muscular stress* (MS), *lack of activation* (LA), *emotional imbalance* (EI) and *overall stress* (OS). For data analysis, mean score for recovery (recovery score (rec); PPC, MPC, EB, OR) and stress (stress (stress) score; MS, LA, EI, OS) were implemented as well as the respective subscales.

Directly after completing the short scale, participants performed a bicycle protocol (LC6 Novo, Monark Exercise AB, Sweden) at the predefined intensity (by means of power) determined in the baseline assessment (90% VT1, see Table 1) and for a duration of ten minutes.

Finally, assessment of effects of the performed exercise session was obtained by the CMJ and Foot Tapping (FT) test on a ground reaction force platform (Leonardo Mechanograph GRFP, Novotec Medical GmbH, Pforzheim, Germany) with associated software (Leonardo Mechanography Software Version 4.3). Three CMJs were carried out according to the specifications of [41] without arm swing. Mean jump height (CMJh) and mean vertical peak force normalized to the participants’ body weight (CMJf) was used for further processing. FT was performed two times in a standing position for 15 s as described by Krauss [42]. Out of these trials, the best one was chosen. Furthermore, number of foot contacts (FTc) and frequency (FTf) were considered for statistical analysis.

### RR measurements and calculation of DFA a1

Single channel ECG.csv files (obtained by the Movesense Medical sensor and exported from the Movesense Showcase app; see Baseline Assessment) were imported into Kubios HRV Premium software Version 3.5.0 (Biosignal Analysis and Medical Imaging Group, Department of Physics, University of Kuopio, Kuopio, Finland, [43]). Preprocessing settings were set to the default values including the RR detrending method which was kept at “smoothness priors” (Lambda = 500). For DFA a1 calculation, the root mean square fluctuation of the integrated and detrended data is measured in observation windows of different sizes. This is done using a logarithmic plot of the data against the size of the window. The resulting slope of the line relating the (log) fluctuation to the (log) window size represents the scaling exponent [44]. DFA a1 window width was set to 4 ≤ n ≤ 16 beats [23]. Artifacts in the RR series were corrected by the Kubios “automatic method” [45] and subject data excluded from further analyses when the overall percent artifact exceeded 5% [46]. To minimize influencing factors at the beginning of the warm-up, DFA a1 and average HR were calculated from the RR data series of the 2-min time window consisting of the start of minute 7 to the end of minute 8 of the cycling exercise on all four occasions. The 2-min window width was chosen based on the minimal beats required for valid DFA a1 calculation [47–49].

### Statistics

Statistical analysis of means and standard deviations (SD) were performed in Microsoft Excel 365, further calculations were done using SPSS 23.0 (IBM Statistics, United States) for Windows (Microsoft, USA). The Shapiro–Wilk test was applied to verify the Gaussian distribution of the data. PRE versus POST differences were respectively analyzed for both conditions (light and heavy exercise session), with a t-test for paired samples in parametric data, whereas nonparametric data was analyzed with a Wilcoxon test for paired samples. Mean differences were specified with the respective 90% confidence interval (90% CI). Statistical significance was accepted at *p* < 0.05. Cohen’s effect sizes were calculated and interpreted as trivial: < 0.2, small: ≤ 0.2 to < 0.5, moderate: ≥ 0.5 to < 0.8, or large: ≥ 0.8 [50]. In addition, magnitude-based inferences were used to report the percentage changes quantitatively (descriptor: negative/trivial/positive) and in relation to the smallest worthwhile change (SWC) as well as qualitatively with indications about clinically relevance [51]. SWC was determined according to the recommendation of Buchheit et al. [14] with the coefficient of variation (CV = SD/Mean) calculated for the whole group at PRE of the light session and multiplied by 0.3 and respective means of performance measures as well as by 0.5 and means of physiological markers. Qualitative interpretations were based on this preset scale: < 0.5% “most unlikely”, 0.5–5% “very unlikely”, 5–25% “unlikely”, 25–75% “possibly”, 75–95% “likely”, 95–99.5 “very likely”, > 99.5% “most likely”. Analyses of the difference between means were performed using the “xcl_Bayesian.xls” spreadsheet obtained from http://sportsci.org/2007/wghinf.htm [52]. Furthermore, individual changes in DFA a1 were assessed based on the typical error of measurement (TE) and SWC using the “xprecision.xls” spreadsheet available on http://sportsci.org/2017/wghtrend.htm [53]. TE was determined according to the recommendations of [54] by calculating the SD of the difference scores from PRE light session and PRE heavy session values and dividing the value by the root of (2). Precision change was specified using 50% CI also based on the reasoning of Swinton et al. [54].

## Results

**Fig. 2:**
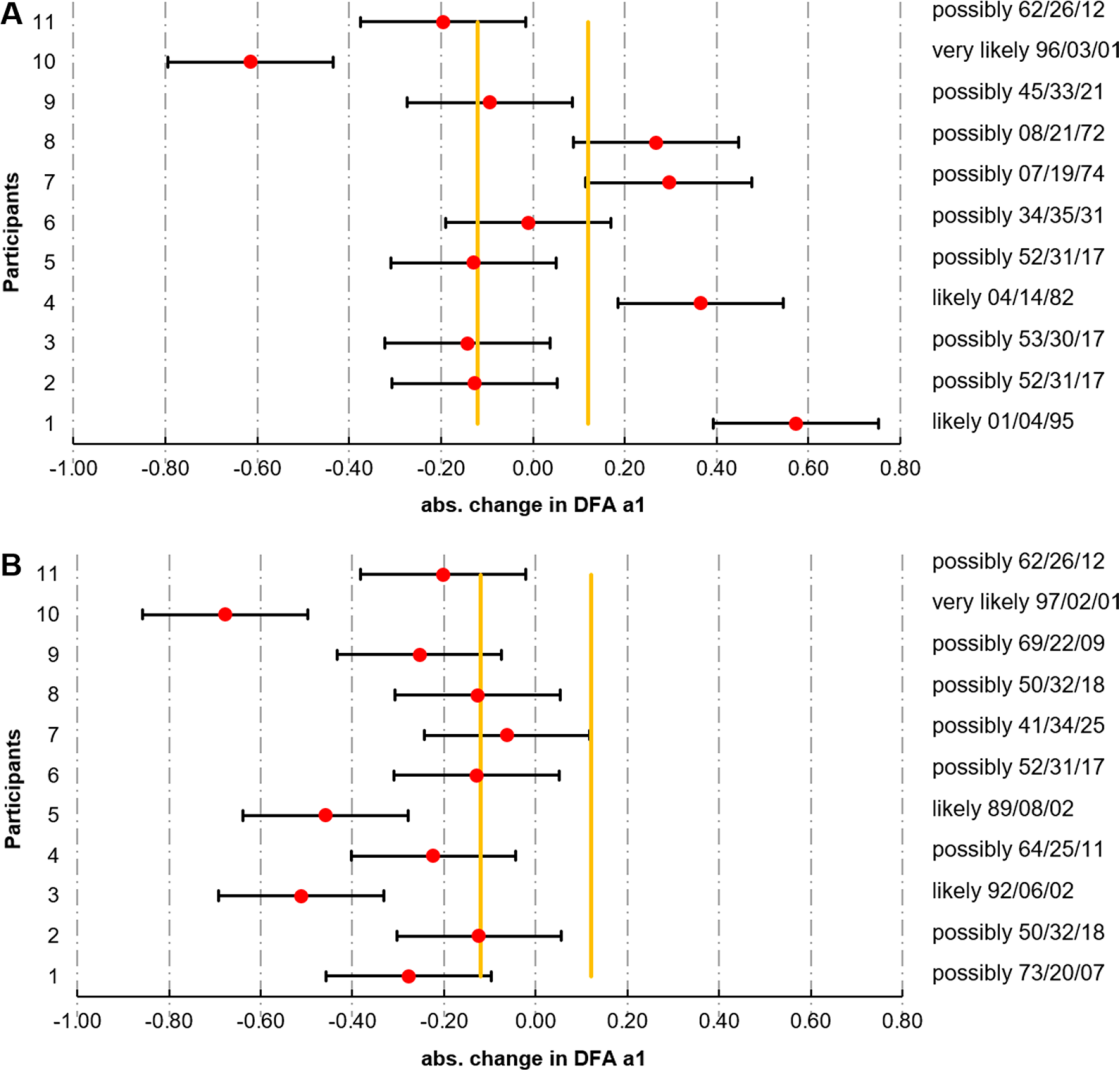
Individual magnitude of change in DFA a1 due to the **A** light and **B** heavy running exercise session for all triathletes. Legend: The absolute differences are depicted by means of confidence intervals (50% CI). The vertical yellow lines mark the smallest worthwhile change representing the area of trivial changes. Percentages and descriptors inform about the individuals’ magnitude of changes in a quantitative and qualitative manner

Mean and SD for the evaluated data can be seen in Tables 3 and 4. RPE was significantly higher for the heavy as opposed to the light running exercise session with large effect size (*p* < 0.001, d = 0.89). With regard to the PRE versus POST comparisons in group means (see Table 5), there were significant reduced DFA a1 values with large effect size after the heavy exercise session (*p* = 0.001, d = − 1.44) and a 99% (“very likely”) chance that this negative change was clinically relevant. Moreover, significant changes could only be found in CMJ vertical peak force (*p* = 0.020, d = 0.84) and FT frequency (*p* = 0.021. d = 0.82) showing higher results following the light exercise session. Although when considering magnitude-based inferences the chances for the true changes were rated “possibly" positive (00/30/70 and 00/65/35, respectively). In addition, inter-individual differences could be observed and the magnitude of change of all participants of DFA a1 due to the respective exercise sessions is depicted in Fig. 2 and is calculated by making use of the determined SWC (0.12) and TE (0.18) listed in Table 5.

**Table 3:** PRE and POST light running exercise session data (n = 11)

	PRE								Unit	POST							
	Rec [0–6]	Stress [0–6]	DFA a1	HR (bpm)	CMJh (cm)	CMJf (N kg^−1^)	FTc	FTf (Hz)	RPE [0–10]	Rec [0–6]	Stress [0–6]	DFA a1	HR (bpm)	CMJh (cm)	CMJf (N kg^−1^)	FTc	FTf (Hz)
1	4.3	2.8	0.63	144	47.0	22.4	148	11.1	3	3.5	3.5	1.20	139	48.9	24.4	152	11.2
2	3.8	1.5	0.83	136	40.5	24.4	117	9.2	2	4.8	0.5	0.70	137	37.8	23.5	124	9.6
3	5.8	0.5	1.32	126	36.2	19.4	108	8.2	3	4.8	1.0	1.18	124	36.4	22.1	104	8.0
4	4.3	2.8	0.77	128	32.6	18.9	122	9.2	3	5.3	0.3	1.13	124	41.0	19.6	126	9.6
5	4.8	0.8	1.25	155	33.8	20.2	150	11.0	2	4.8	1.0	1.12	158	34.7	20.4	160	11.9
6	3.3	3.0	0.84	147	38.3	22.7	96	7.3	2	3.4	2.8	0.83	137	38.2	23.9	104	8.0
7	4.8	1.5	0.93	121	40.6	23.2	146	10.9	3	4.8	1.3	1.22	117	43.2	24.4	148	11.0
8	4.0	2.0	0.81	122	36.3	17.9	112	8.5	3	3.8	1.5	1.08	122	36.5	17.8	120	9.2
9	3.1	2.5	0.93	116	36.2	18.4	110	8.5	2	2.9	1.0	0.84	123	36.8	18.6	118	9.0
10	4.0	2.3	1.36	160	36.9	18.6	138	10.4	3	3.5	2.3	0.75	170	35.5	19.2	134	11.0
11	4.0	3.0	0.96	131	36.2	18.6	124	9.4	3	3.8	3.8	0.76	142	37.0	20.2	120	9.0
Mean ± SD	4.2 ± 0.7	2.1 ± 0.9	0.97 ± 0.24	135 ± 15	37.7 ± 3.9	20.4 ± 2.3	125 ± 18	9.4 ± 1.3	3 ± 0	4.1 ± 0.8	1.7 ± 1.2	0.98 ± 0.21	136 ± 16	38.7 ± 4.2	21.3 ± 2.5	128 ± 19	9.8 ± 1.3

rec = mean score for recovery; stress = mean score for stress; DFA a1: short-term scaling exponent alpha 1 of Detrended Fluctuation Analysis; HR = heart rate; CMJh = mean jump height; CMJf = mean vertical peak force normalized to body mass; FTc = Foot Tapping contacts; FTf = Foot Tapping frequency; RPE = rating of perceived exertion

**Table 4:** PRE and POST heavy running exercise session data (n = 11)

	PRE								Unit	POST							
	Rec [0–6]	Stress [0–6]	DFA a1	HR [bpm]	CMJh [cm]	CMJf [N kg^−1^]	FTc	FTf [Hz]	RPE [0–10]	Rec [0–6]	Stress [0–6]	DFA a1	HR [bpm]	CMJh [cm]	CMJf [N kg^−1^]	FTc	FTf [Hz]
1	4.5	3.8	1.45	151	49.3	22.9	160	11.8	5	3.0	2.0	1.17	142	47.4	24.5	152	11.2
2	3.8	1.5	0.74	133	41.2	22.7	126	9.5	8	3.8	2.5	0.62	131	39.2	22.3	132	10.0
3	5.3	0.8	1.43	128	35.8	19.5	112	8.7	8	5.5	0.5	0.92	127	35.8	20.0	122	9.3
4	5.5	0.3	0.76	126	33.9	21.4	138	10.4	5	4.5	2.0	0.54	125	38.9	22.2	134	10.2
5	4.0	1.5	1.40	147	34.3	19.7	156	11.6	7	3.8	1.8	0.94	147	31.1	20.6	138	11.5
6	3.4	2.5	1.32	121	36.5	23.7	113	8.7	7	3.1	1.8	1.19	128	41.2	23.3	125	9.4
7	3.1	1.5	1.21	120	40.0	24.2	138	10.2	8	3.1	1.8	1.15	118	42.7	23.2	147	11.0
8	3.1	1.8	1.02	124	35.4	17.6	124	9.5	7	3.1	2.0	0.90	119	38.3	17.7	117	8.9
9	3.5	2.8	1.18	125	34.8	18.6	126	9.6	8	3.0	2.5	0.93	116	34.9	19.3	132	10.0
10	3.1	2.3	1.47	154	36.5	19.3	124	9.3	7	3.3	2.3	0.79	160	36.8	19.1	145	10.8
11	3.1	3.3	0.93	130	-	-	124	9.4	8	3.1	2.5	0.73	133	-	-	122	9.3
Mean ± SD	3.9 ± 0.9	2.0 ± 1.1	1.17 ± 0.27	133 ± 12	37.8 ± 4.7	21.0 ± 2.3	131 ± 16	9.9 ± 1.0	7 ± 1	3.6 ± 0.8	2.0 ± 0.6	0.90 ± 0.22	131 ± 13	38.6 ± 4.5	21.2 ± 2.2	133 ± 11	10.1 ± 0.9

rec, mean score for recovery; stress, mean score for stress; DFA a1, short-term scaling exponent alpha 1 of Detrended Fluctuation Analysis; HR, heart rate; CMJh, mean jump height; CMJf, mean vertical peak force normalized to body mass; FTc, Foot Tapping contacts; FTf, Foot Tapping frequency; RPE, rating of perceived exertion

**Table 5:** Statistical comparison of the measures for PRE and POST conditions respectively

	Rec [0–6]	Stress [0–6]	DFA a1	HR (bpm)	CMJh (cm)	CMJf (N kg^−1^)	FTc	FTf (Hz)
**Statistical reference data**								
TE (absolute)	0.57	0.64	0.18	6.58	7.75	4.11	7.20	0.57
CV (%)	18	43	25	11	10	11	15	13
SWC (absolute)	0.37	0.44	0.12	7.33	1.18	0.69	5.50	0.38
**PRE-POST change light running exercise session**								
Mean difference (90% CI)	− 0.07 ± 0.12 (− 0.19–0.05)	− 0.34 ± 0.47 (− 0.81–0.13)	0.02 ± 0.18 (− 0.16–0.20)	0.51 ± 3.67 (− 3.16–4.18)	1.04 ± 1.03 (0.00–2.07)	0.85 ± 0.56 (0.30–1.41)	3.55 ± 2.93 (0.62–6.47)	0.33 ± 0.22 (0.11–0.55)
*p* value	0.291	0.223	0.866	0.805	0.099	0.020	0.053	0.021
Cohen's d	− 0.20	− 0.25	0.05	0.08	0.40	0.84	0.66	0.82
**Cohen's d descriptor**	Small	Small	Trivial	Trivial	Small	Large	Moderate	Large
% change Negative/trivial/positive (descriptor)	00/100/00 Most likely trivial	35/64/01 Possibly trivial	10/74/16 Possibly trivial	00/100/00 Very likely trivial	00/60/40 Possibly positive	00/30/70 Possibly positive	00/87/13 Likely trivial	00/65/35 Possibly positive
*PRE-POST change heavy running exercise session*								
Mean difference (90% CI)	− 0.28 ± 0.24 (− 0.53–0.04)	− 0.04 ± 0.08 (− 0.11–0.04)	− 0.28 ± 0.11 (− 0.38-(− 0.17))	− 1.40 ± 2.89 (− 4.30–1.49)	0.86 ± 1.65 (− 0.79–2.51)	0.26 ± 0.45 (− 0.19–0.71)	2.27 ± 6.08 (− 3.81–8.35)	0.25 ± 0.35 (− 0.09–0.60)
*p* value	0.063	0.414	0.001	0.400	0.366	0.318	0.514	0.214
Cohen's d	− 0.51	− 0.08	− 1.44	− 0.27	0.30	0.33	0.20	0.40
Cohen's d descriptor	Moderate	Trivial	Large	Small	Small	Small	Small	Small
% change Negative/trivial/positive (descriptor)	26/74/00 Possibly trivial	00/100/00 Most likely trivial	99/01/00 Very likely negative	00/100/00 Most likely trivial	03/61/36 Unclear	00/94/06 Likely trivial	02/80/18 Likely trivial	00/74/26 Possibly positive

rec, mean score for recovery; stress, mean score for stress; DFA a1, short-term scaling exponent alpha 1 of Detrended Fluctuation Analysis; HR, heart rate; CMJh, mean jump height; CMJf, mean vertical peak force normalized to body mass; FTc, Foot Tapping contacts; FTf, Foot Tapping frequency; TE, typical error; CV, coefficient of variation; SWC, smallest worthwhile change. Data is expressed as mean differences (90% confidence intervals (90% CI), *p* value, standardized mean difference (Cohen’s d and descriptor) and as the percentage change with clinically relevance (percentages and descriptor)

## Discussion

This study examined whether DFA a1 behavior during a low intensity warm-up cycling session could be used to assess an altered physiological status as a surrogate for “readiness to train” and thus guide athletes in their training routine as an easy-to-apply monitoring procedure. In accordance with the findings of Rogers et al. [21], this study indicates a relative suppression of DFA a1 during low intensity exercise, even up to 36 h post exercise. Magnitude-based inferences indicated that suppression of DFA a1 in POST heavy exercise session (99% negative chance) was “very likely” clinically relevant, while alterations in HR and psychometrics were interpreted as “trivial” (74–100% chance). Performance measures demonstrated similar qualitative descriptors as HR and psychometrics in response to the heavy exercise session with no significant change, although showing possibly positive alterations which could only be affirmed in FTf. Interestingly, psychometrics and both HR and DFA a1 showed no sensitivity in response to the light exercise session (64–100% “trivial” chance), while changes in CMJh, CMJf and FTf illustrated a higher likelihood of being clinically positive (35–70% chance). However, significance was only attained in CMJf and FTf.

With respect to the explanations of the “readiness” construct from the introduction, this state was maintained after both training sessions pointing to an absence of fatigue due to no deterioration in neuromuscular function (CMJ, FT) nor SRSS (by definition). However, since practicability of neuromuscular function assessments is challenged by contextual and individual elements (e.g. motivation, familarization, physical qualities, season stage) that may undermine inferences derived from the data [31] as well as varying sensitivity of the metrics depending on the time course of the effect [10], it becomes clear that decision-making based solely on (neuromuscular) performance metrics may not always be correct. Taking a deeper look on the analysis of the SRSS subscales (not included in the tables for reasons of clarity), OR did show a significant decrease when comparing PRE and POST values of the heavy exercise session with moderate effect size (*p* = 0.031, d = − 0.62). The participants felt less refreshed, rested, muscularly loose and physically relaxed after the heavy running exercise session performed. This is probably also reflected in DFA a1 since this metric is assumed to be a proxy of autonomic balance and organismic demands [25].

Standardized submaximal exercise assessments are very often used to monitor physiological status with HR-derived indices being the most studied outcome measure [31]. However, they seem to detect especially positive chronic endurance-oriented training effects rather than negative transient effects associated with variations in autonomic nervous system function [31]. Although it is a relatively novel concept to use DFA a1 as an outcome measure during a standardized submaximal exercise session, the findings of the existing studies to date [21, 55] are already used by a web based application (AI endurance: https://aiendurance.com/) for the purpose of training monitoring. This training tool assumes that a suppressed DFA a1 from a baseline at a given power or pace indicates that an individual is not performing well and not ready to train or race with high exercise intensity and/or volume [21]. In support of this concept, although there was a drop in DFA a1 for all triathletes after the heavy exercise session indicating a loss of correlation properties of HR time series (and a disturbance of the ANS regulation), some showed greater declines than others, pointing to highly individualized responses to the exercise intensity performed. It is therefore crucial to observe the extent of changes in an individual athlete (for example by means of magnitude-based inferences shown for all participants in Fig. 2), rather than to derive general tendencies or statistical significance of the change. This can be illustrated by examining two example cases: Both participants 4 and 7 showed “likely” and “possibly” PRE versus POST increases in DFA a1 with light exercise session (82 and 74% positive chance respectively). However, the comparison regarding heavy exercise session revealed deviating trends: While the change of participant 7 remained mostly within the determined SWC and values of DFA a1 were still between the range of 1.0 to 1.5, there was a possible decrease (64% negative chance) observed to a value near the anti-correlated range (< 0.5) in participant 4 pointing to a physiologic fatigue status and potential metabolic destabilization [21, 56]. Therefore, it may be reasonable to recommend a decreased exercise load for the upcoming session as a result of the abnormal autonomic response of athlete 4 during the warm-up. For participant 7 training could be continued in the intended way (proceed as planned). With the implementation of this procedure on a regular basis, it may be possible to capture not only acute status, but performance improvements or load tolerance when observing DFA a1 kinetics in regard to different exercise and training interventions. However, this should be investigated in further studies with larger and more homogeneous populations.

In context of the mentioned NPE approach, a reduction of the human organism to any single component is incomplete as the synchronization and interaction of components generates novel information which determines the function of subelements and of the system itself [30]. Since DFA a1 is suspected to be capable to quantify this dynamic network interactions in regards of ANS regulation, it would seem obvious to designate this metric as a biomarker to assess the physiological status as a surrogate metric for “readiness to train”. Nevertheless, the current conceptualization of the term “readiness to train” is highly related to neuromuscular function metrics which has not changed in comparison to DFA a1 in the present data. We therefore hypothesize DFA a1 to be better suitable to reflect the overall physiological status rather than to just depict one measure of a physiological subsystem.

### Limitations and future directions

This study focused on the assessment of the physiological status as a surrogate metric for “readiness to train” following a light and heavy running exercise session. Although an acute estimation could be given, it would have been interesting to know the broader training context of the participants which has been not assessed and analyzed. It was not known if there were some triathletes experiencing an overreached, non-overreached or even overtrained state since this could further elucidate potential individual deviations. Additionally, internal load of exercise sessions performed was only quantified by RPE. It would have been useful to capture the internal load in more detail to assure that the sessions were executed according to the prescription and to minimize potential bias in the triathletes’ ratings due to the knowledge of their coaches’ training conception. Future work should account for optimized methodological considerations with regard to the implemented tests, evaluation methods or familiarization periods, so that a fatigued state can be assumed with high certainty. A familiarization and baseline period for some of the metrics could serve for establishing statistical reference data (TE, CV, SWC) for the test measures used a prori. This study calculated CV and SWC from the group values of PRE light exercise session and TE using PRE light and PRE heavy exercise session data from all participants where true scores are not expected to change [54]. Due to the small sample size and low number of repeated tests, the obtained values for TE, CV and SWC are expected to be more accurate with a range of intra-individual data, potentially affecting the results obtained from the magnitude-based inferences. With regard to DFA a1, an individual could obtain better insights by examining their personal SWC, CV and TE during the same exercise protocol [54]. In general, this statistical approach can also help practitioners in decision-making for individual monitoring, irrespective of the parameter used.

Currently, there are real-time approaches available for smartphone application (e.g. Fatmaxxer: https://github.com/IanPeake/FatMaxxer) providing continuous monitoring with a DFA a1 recalculation every 5–30 s (user defined). This provides the opportunity to apply the described monitoring approach in the warm-up period to get a quick snapshot of the physiological status. It would certainly be worthwhile if there were software options to save historical data, thus creating a baseline that corresponds to the SWC corridor (as already used in a similar manner in the already mentioned AI endurance application). With regular data collection, more accurate assessments could be made, which would potentially allow the user to better manage their exercise and training intensity distribution.

## Conclusion

Observation of the DFA a1 to power/pace relationship during simulated warm-up bouts (performed PRE- and within 36 h POST-exercise at both low and high demand) seems to represent a valuable measure to depict physiological status in this heterogenous group of triathletes. In the same manner as monitoring resting HRV physiology, it may be possible to utilize inappropriate suppression of DFA a1 at low exercise intensity as a submaximal test and as a means of daily directed training. A regular assessment including individual data history and statistical references for identification of response (change in true score exceeds the smallest worthwhile change, and a normal intra-individual range based on historical data) is recommended. Further data are necessary to confirm the results in a larger and more homogeneous population.

## Data Availability

The raw data generated during the current study are available in the OSF Data repository: https://osf.io/fjqnk/.
