# Correction: Fractal correlation properties of HRV as a noninvasive biomarker to assess the physiological status of triathletes during simulated warm-up sessions at low exercise intensity: a pilot study

**DOI:** 10.1186/s13102-022-00602-2

**Published:** 2022-12-12

**Authors:** Marcelle Schafarczyk, Bruce Rogers, Rüdiger Reer, Thomas Gronwald

**Affiliations:** 1 Institute of Interdisciplinary Exercise Science and Sports Medicine, MSH Medical School Hamburg, University of Applied Sciences and Medical University, Am Kaiserkai 1, 20457 Hamburg, Germany; 2 Department of Internal Medicine, College of Medicine, University of Central Florida, Orlando, USA; 3 Department Sports and Exercise Medicine, Institute of Human Movement Science, University of Hamburg, Hamburg, Germany

**Correction: BMC Sports Science, Medicine and Rehabilitation (2022) 14:203** **https://doi.org/10.1186/s13102-022-00596-x**

Following publication of the original article [1], the authors identified a typesetting error in Table 5. The bold subheadings in column 1 were incorrectly formatted. The corrected Table 5 is published in this correction article and the original article [1] has been corrected.

**Table 5:** Statistical comparison of the measures for PRE and POST conditions respectively

	Rec [0–6]	Stress [0–6]	DFA a1	HR (bpm)	CMJh (cm)	CMJf (N kg^−1^)	FTc	FTf (Hz)
**Statistical reference data**								
TE (absolute)	0.57	0.64	0.18	6.58	7.75	4.11	7.20	0.57
CV (%)	18	43	25	11	10	11	15	13
SWC (absolute)	0.37	0.44	0.12	7.33	1.18	0.69	5.50	0.38
**PRE-POST change light running exercise session**								
Mean difference (90% CI)	− 0.07 ± 0.12 (− 0.19–0.05)	− 0.34 ± 0.47 (− 0.81–0.13)	0.02 ± 0.18 (− 0.16–0.20)	0.51 ± 3.67 (− 3.16–4.18)	1.04 ± 1.03 (0.00–2.07)	0.85 ± 0.56 (0.30–1.41)	3.55 ± 2.93 (0.62–6.47)	0.33 ± 0.22 (0.11–0.55)
*p* value	0.291	0.223	0.866	0.805	0.099	0.020	0.053	0.021
Cohen's d	− 0.20	− 0.25	0.05	0.08	0.40	0.84	0.66	0.82
Cohen's d descriptor	Small	Small	Trivial	Trivial	Small	Large	Moderate	Large
% change Negative/trivial/positive (descriptor)	00/100/00 Most likely trivial	35/64/01 Possibly trivial	10/74/16 Possibly trivial	00/100/00 Very likely trivial	00/60/40 Possibly positive	00/30/70 Possibly positive	00/87/13 Likely trivial	00/65/35 Possibly positive
**PRE-POST change heavy running exercise session**								
Mean difference (90% CI)	− 0.28 ± 0.24 (− 0.53–0.04)	− 0.04 ± 0.08 (− 0.11–0.04)	− 0.28 ± 0.11 (− 0.38-(− 0.17))	− 1.40 ± 2.89 (− 4.30–1.49)	0.86 ± 1.65 (− 0.79–2.51)	0.26 ± 0.45 (− 0.19–0.71)	2.27 ± 6.08 (− 3.81–8.35)	0.25 ± 0.35 (− 0.09–0.60)
*p* value	0.063	0.414	0.001	0.400	0.366	0.318	0.514	0.214
Cohen's d	− 0.51	− 0.08	− 1.44	− 0.27	0.30	0.33	0.20	0.40
Cohen's d descriptor	Moderate	Trivial	Large	Small	Small	Small	Small	Small
% change Negative/trivial/positive (descriptor)	26/74/00 Possibly trivial	00/100/00 Most likely trivial	99/01/00 Very likely negative	00/100/00 Most likely trivial	03/61/36 Unclear	00/94/06 Likely trivial	02/80/18 Likely trivial	00/74/26 Possibly positive

rec, mean score for recovery; stress, mean score for stress; DFA a1, short-term scaling exponent alpha 1 of Detrended Fluctuation Analysis; HR, heart rate; CMJh, mean jump height; CMJf, mean vertical peak force normalized to body mass; FTc, Foot Tapping contacts; FTf, Foot Tapping frequency; TE, typical error; CV, coefficient of variation; SWC, smallest worthwhile change. Data is expressed as mean differences (90% confidence intervals (90% CI), *p* value, standardized mean difference (Cohen’s d and descriptor) and as the percentage change with clinically relevance (percentages and descriptor)
